# Warfarin-induced spontaneous intramural small bowel hematoma presenting as an acute abdomen: A case report

**DOI:** 10.1097/MD.0000000000030319

**Published:** 2022-09-02

**Authors:** Ding-Han Chen, Khay-Seng Soh, Ying-Tso Wang, Te-Chun Shen

**Affiliations:** a Division of Pulmonary and Critical Care Medicine, Department of Internal Medicine, China Medical University Hospital, Taichung, Taiwan; b Department of Surgery, China Medical University Hospital, Taichung, Taiwan; c Department of Surgery, Chu Shang Show Chwan Hospital, Nantou, Taiwan; d Department of Pathology, China Medical University Hospital, Taichung, Taiwan; e Department of Critical Care Medicine, Chu Shang Show Chwan Hospital, Nantou, Taiwan; f School of Medicine, China Medical University, Taichung, Taiwan.

**Keywords:** acute abdomen, anticoagulation, case report, intestinal obstruction, spontaneous intramural small bowel hematoma (SISBH), warfarin

## Abstract

Spontaneous intramural small-bowel hematoma (SISBH) is a rare complication of anticoagulation therapy. Presentation of SISBH can vary from mild abdominal pain to an acute abdomen.

**Patient concerns:**

A 70-year-old woman was brought to the emergency department because of severe abdominal pain for 1 day. She had a medical history of coronary artery disease and paroxysmal atrial fibrillation and was receiving anticoagulation therapy with warfarin for 3 years.

**Diagnosis:**

Computed tomography disclosed disproportional dilatation of the segmental small bowel and near-total obstruction of the intestinal lumen at the level of the jejunum, indicating an acute abdomen.

**Interventions:**

We performed laparoscopic exploration and found a segmental distal jejunum was tense, heavy, firm, and discolored with a blue hue. Histopathological examination of the resected jejunum revealed diffuse hemorrhage and necrosis at the mucosa and submucosal layers, indicating SISBH.

**Outcomes:**

The patient had an uneventful recovery and was discharged in a relatively stable condition.

**Lessons:**

Warfarin-induced SISBH presenting as an acute abdomen is an emergency condition that needs early diagnosis and timely management. Surgical intervention may be indicated for intestinal obstruction, ischemia, perforation, peritonitis, and intra-abdominal hemorrhage.

## 1. Introduction

Anticoagulation therapy is widely applied for various thromboembolic and coagulation disorders. It could trigger various adverse events, with bleeding as the most common. The incidence of bleeding complications of anticoagulation therapy ranges from 5% to 48%, but gastrointestinal bleeding occurs in only 2%–4% of cases.^[1]^ Spontaneous intramural small-bowel hematoma (SISBH) is a rare complication of anticoagulation therapy. It occurs only in approximately 1 of 2500 patients receiving anticoagulation therapy.^[2]^ However, the incidence is expected to increase; thus, awareness for this entity is important. Herein, we presented a 70-year-old woman who developed severe abdominal pain due to warfarin-induced SISBH. The patient has provided informed consent for the publication of the case.

## 2. Case presentation

A 70-year-old woman was brought to the emergency department because of severe abdominal pain for 1 day. She had a medical history of coronary artery disease and paroxysmal atrial fibrillation and was receiving anticoagulation therapy with warfarin 2.5 mg per day for 3 years. The international normalized ratio level was under adequate control at around 2–3.

Physical examination on arrival showed body temperature, 37.4°C; pulse, 108 beats/min; respiration, 20 breaths/min; blood pressure, 138/76 mmHg, abdominal distension, diffuse tenderness, and rebounded tenderness. Laboratory data revealed increased white blood cell counts with a neutrophil predominance (24,030/µL, 89%), decreased hemoglobin level (9.6 g/L), normal platelet count (228,000/µL), prolonged prothrombin time (>80 s), and prolonged activated partial thromboplastin time (111.9 s).

**Figure 1.**
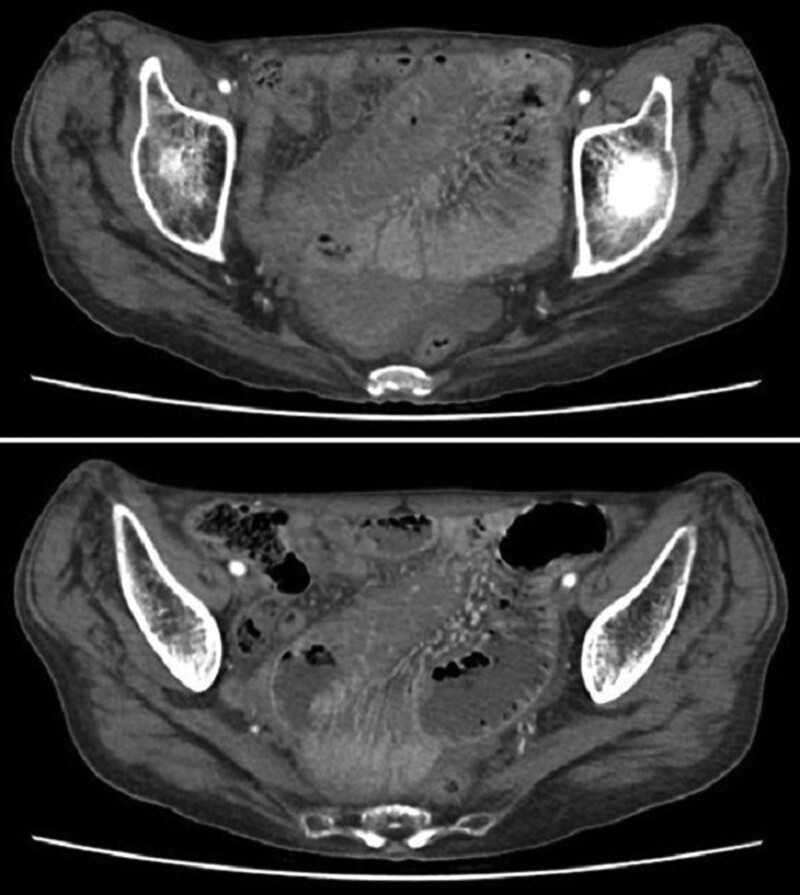
Computed tomography disclosed disproportional dilatation of the segmental small bowel, poorly enhanced mucosal circulation with severely swollen mucosa and near-total obstruction of the intestinal lumen at the level of the jejunum.

**Figure 2.**
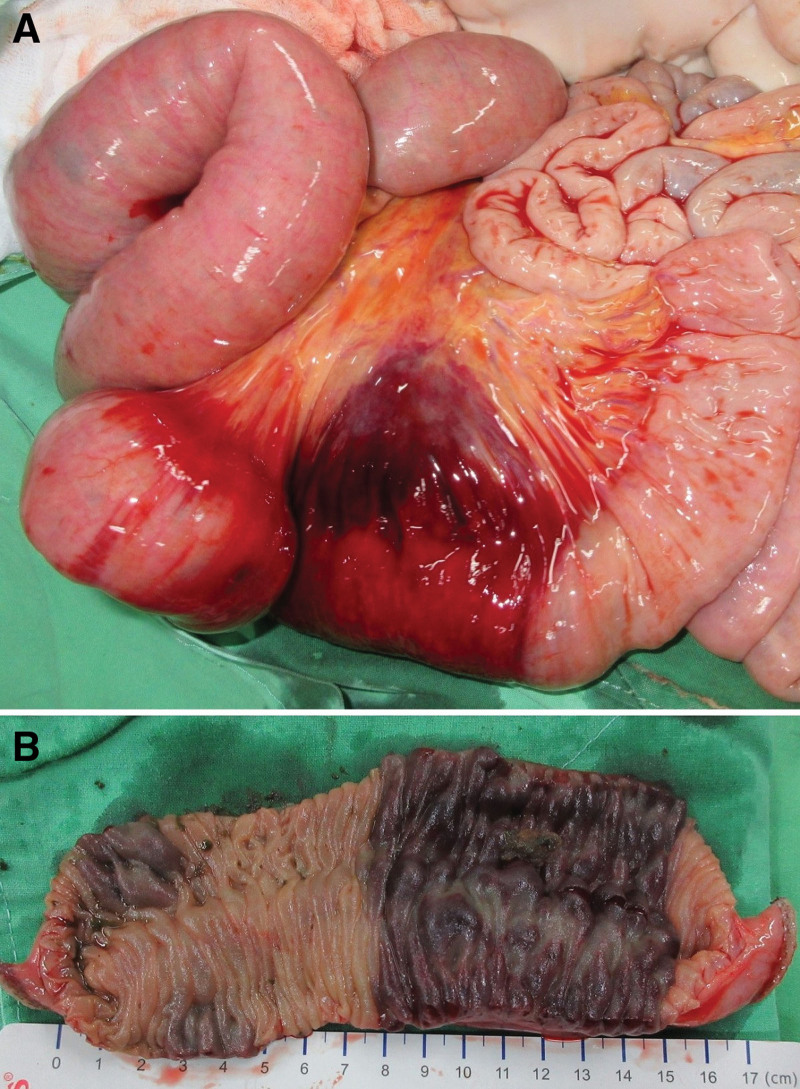
(A) A segmental jejunum was found to be tense, heavy, firm, and discolored with a blue hue. (B) The specimen was dissected, which showed an intramural hematoma with near-total obstruction of the intestinal lumen.

Emergency computed tomography (CT) disclosed disproportional dilatation of the segmental small bowel, poorly enhanced mucosal circulation with severely swollen mucosa, and near-total obstruction of the intestinal lumen at the level of the jejunum (Fig. 1). Laparoscopic exploration was thus performed, and bloody ascites was observed. A segmental distal jejunum (7 cm in length) was tense, heavy, firm, and discolored with a blue hue, and multiple dilated loops of the small bowel were found proximal to this lesion (Fig. 2A). Swollen and congested small-bowel mesentery was also noted, which probably contributed to the poor circulation of the involved jejunum. Segmental resection and primary anastomosis were thus performed smoothly. The blood loss during the operation was about 100 mL and the patient received fresh frozen plasma 4 units (500 mL) transfusion to correct the coagulopathy encountered during the operation. The specimen was dissected and showed an intramural hematoma with near-total obstruction of the intestinal lumen (Fig. 2B).

Histopathological examination of the resected jejunum did not reveal the presence of a thrombus in mesentery vessels. Diffuse hemorrhage and necrosis were found at the mucosa and submucosal layers. Thus, the patient was diagnosed with warfarin-induced SISBH. The patient had an uneventful recovery and was discharged in a relatively stable condition. The cardiologist suggested the use of apixaban (5 mg twice a day) after discharge. No further adverse drug reactions or cardiovascular events were noticed during the 12-month follow-up.

## 3. Discussion

SISBH is a rare complication of anticoagulation therapy, with warfarin as the most common causative agent. The presentation of SISBH can vary from mild and vague abdominal pain to intestinal obstruction and an acute abdomen. Warfarin-induced SISBH presenting as an acute abdomen is a very rare condition. Conservative treatment was commonly applied; surgical intervention may be reserved for severe conditions, such as intestinal obstruction, ischemia, perforation, peritonitis, and intra-abdominal hemorrhage.^[3]^

Abbas et al^[4]^ first analyzed 13 cases of SISBH between 1983 and 2000 in the United States. They reported that a single hematoma was present in 85% of the patients, and multiple hematomas in 15%. The jejunum was the most commonly involved site (69%), followed by the ileum (38%) and duodenum (23%). CT findings included circumferential wall thickening, intramural hyperdensity, luminal narrowing, and intestinal obstruction. The mortality rate was 15%.

Subsequently, Altintoprak et al^[5]^ collected 15 cases of SISBH between 2007 and 2011 from 3 hospitals in Turkey. They reported that the initial sites of medical visits were emergency departments for 10 (66.6%) patients and clinics for 5 (33.3%) patients. The most common symptom was abdominal pain (100%), followed by vomiting (53.3%), weakness (40%), and anorexia (26.6%). Single and multiple hematomas were noted in 73.3% and 26.7% of the patients, respectively. Thirteen patients (86.6%) received medical treatment, and 2 (13.3%) underwent surgical treatment. The mortality rate was 13.3%.

Recently, Kang et al^[6]^ identified 37 cases of SISBH between 2003 and 2016 from 2 tertiary hospitals in Korea. They were located in the ileum (45.9%), jejunum (43.2%), and duodenum (10.8%). The initial symptoms were abdominal pain (64.9%), gastrointestinal bleeding (37.8%), and nausea and/or vomiting (35.1%). All patients were treated successfully with conservative therapy including fasting, blood component transfusion, and/or vitamin K supplement. The mean length of hospital stay was 9.35 days.

A recent literature review of 40 articles within the previous 30 years analyzed a total of 103 cases of SISBH.^[6]^ The mean age of the patients was 62.2 years, and abdominal pain (72.8%) was the most frequent symptom. Anticoagulant and antiplatelet therapies (78.6%) were the most common cause of SISBH. Among these medications that induce SISBH, warfarin (73.8%) was the most frequently associated agent. Common risk factors for SISBH may include cardiovascular diseases, hemophilia, pancreatic diseases, liver failure, leukemia, idiopathic thrombocytopenic purpura, and lupus. However, the risk factors for warfarin-induced SISBH remain uncertain. The involved sites were the jejunum (37.9%), ileum (24.3%), and duodenum (17.5%). Of the 103 patients with SISBH, 18 (17.5%) required surgical treatment for peritonitis, intestinal obstruction, or necrotizing pancreatitis. In other words, SISBH could be commonly treated with conservative therapy. The surgical indications should, however, be further evaluated and identified. The conservative therapy includes fasting, blood component transfusion, and/or vitamin K injection. Only 6 deaths (5.8%) occurred, and the causes of death were sepsis or multiorgan failure.

Warfarin-induced SISBH presenting as an acute abdomen is a very rare condition. It should be included in the differential diagnoses of acute abdomen, particularly in patients undergoing anticoagulation therapy. Early diagnosis and adequate treatment could contribute to a better prognosis.

## Author contributions

Conceptualization: KSS, TCS.

Data curation: DHC, KSS, YTW, TCS.

Formal analysis: DHC, KSS, TCS.

Investigation: KSS.

Writing—original draft: DHC, KSS, YTW, TCS.

Writing—review & editing: DHC, KSS, TCS.
